# Readability of AI-Generated Patient Visit Summaries in Orthopedic Surgery: Retrospective Analysis

**DOI:** 10.2196/87283

**Published:** 2026-08-20

**Authors:** Edward Lee Major III, Vivek P Shah, Amber N Carroll, James R Goetz, Chaitu Malempati, Sameer Badarudeen

**Affiliations:** 1 Department of Orthopaedic Surgery and Sports Medicine, College of Medicine, University of Kentucky, 740 S Limestone St, Lexington, KY, United States, 1 323 873 4940; 2 Department of Orthopaedic Surgery, McLaren Regional Medical Center, Flint, MI, United States; 3 Department of Orthopaedic Surgery, Indiana University, Indianapolis, IN, United States

**Keywords:** artificial intelligence, patient education materials, patient visit summary, readability, health literacy, AI

## Abstract

**Background:**

Patient visit summaries (PVS) are patient-facing documents intended to reinforce communication and promote patient education after clinical encounters. Despite national recommendations that patient education materials be written at or below a sixth-grade reading level, most orthopedic materials substantially exceed this threshold. Current visit summaries are also time-consuming to generate, lack personalization, and often fail to meet patient literacy needs. AI-based scribes can generate personalized PVS in real time directly from patient-provider conversations, offering a potential solution.

**Objective:**

This study aimed to evaluate the readability of AI-generated PVS produced by a commercial AI scribe platform in an orthopedic surgery setting and determine their alignment with established literacy standards for patient-facing materials.

**Methods:**

A total of 1007 consecutive AI-generated PVS from an academic orthopedic surgery outpatient clinic between December 2023 and May 2024 were reviewed. Following standardized preprocessing, including restoration of original section headings and removal of diagnosis label headers, summaries identified as incomplete were excluded (n=25), yielding a final study cohort of 982 summaries. Readability was assessed using 5 validated indices: the Flesch-Kincaid Grade Level (FKGL), Flesch Reading Ease Score (FRES), Gunning Fog Index (GFI), Coleman-Liau Index (CLI), and Simple Measure of Gobbledygook (SMOG) Index. The proportions of PVS meeting the sixth- and eighth-grade benchmarks were calculated. Spearman’s rank correlation (ρ) assessed associations between word count and readability metrics. The Kendall Coefficient of Concordance (*W*) was used to evaluate agreement among indices after reverse-coding FRES for directional alignment. Statistical significance was set at *P*<.05.

**Results:**

Among 982 analyzed PVS, the mean FKGL was 9.3 (SD 1.2, 95% CI 9.2‐9.4) and the mean FRES was 57.6 (SD 7.9, 95% CI 57.1‐58.1), corresponding to “fairly difficult.” Mean scores for secondary indices were 12.4 (SD 1.6) for GFI, 11.0 (SD 1.5) for CLI, and 12.4 (SD 1.2) for SMOG. Only 0.4% (4/982) of PVS met the sixth-grade benchmark and 14.2% (139/982) met the eighth-grade threshold. Word count was not significantly correlated with FKGL (ρ=0.012, *P*=.70), suggesting that sentence structure and vocabulary rather than document length drive reading complexity. Readability indices demonstrated strong agreement across all 5 metrics (*W*=0.882, *P*<.001).

**Conclusions:**

In this orthopedic outpatient setting, AI-generated PVS consistently exceeded recommended patient literacy thresholds, with a mean ninth-grade reading level and only 14.2% of summaries meeting the eighth-grade standard. In the absence of a concurrent comparison group, the present study was not designed to evaluate improvement over traditional methods. These findings establish a quantitative readability baseline for AI scribe output in orthopedic surgery and highlight the need for algorithmic refinement, plain language optimization, and prospective patient comprehension testing to improve health communication.

## Introduction

Effective communication between health care providers and patients is essential for achieving optimal health outcomes [1,2]. Although the average US adult reads at the eighth-grade level, a substantial proportion of patients have lower literacy skills and may struggle to comprehend medical information discussed during clinical encounters [3]. Studies have shown that patients frequently forget or misinterpret key details, which can negatively affect treatment adherence, satisfaction, and safety [4,5]. Recognizing these challenges, national organizations such as the American Academy of Orthopedic Surgeons have recommended that patient education materials be written at or below a sixth-grade reading level [5,6]. However, most orthopedic patient education materials are written above the recommended reading levels, limiting their utility for many patients and providers [7-9].

Providing educational materials and personalized visit summaries after clinical encounters is a standard practice intended to support patient understanding and engagement [10,11]. These summaries are typically generated through manual entry, standardized templates, or automated extraction from electronic health records. However, each method has limitations: manual entry is time-consuming and may be inconsistently applied [12]; templates often lack personalization and may include irrelevant or generic content [13]; and automated extraction can result in inaccuracies, formatting inconsistencies, and limited customization for individual patient needs [14,15]. These challenges often result in summaries that are difficult for patients to comprehend, highlighting the need for innovative strategies that enhance readability, personalization, and accessibility for diverse patient populations [11,16].

AI platforms such as ambient scribes and generative language models can rapidly and accurately convert complex educational materials to recommended readability levels while maintaining content quality [2,9]. AI-based scribe platforms, such as the commercial Abridge system evaluated in this study, generate both structured clinical documentation and personalized visit summaries. Unlike traditional methods, AI tools use natural language processing and real-time audio recordings of patient-provider conversations to generate dynamic summaries that simplify medical jargon and integrate patient-specific concerns, potentially addressing the limitations of current materials [9,17,18]. However, the readability of these summaries remains unexplored in the field of orthopedics, where health literacy has been shown to be lower than that in the general medical population [8].

This study assessed the readability of patient visit summaries (PVS) produced by an AI-based scribe in an outpatient orthopedic clinic. The primary objectives were to determine the baseline readability of these AI-generated summaries and their alignment with the national literacy recommendations. We hypothesized that AI-generated summaries would demonstrate improved readability and closer alignment with recommended literacy standards for patient-facing materials, potentially helping address long-standing communication barriers. By situating these tools within the broader evolution of clinical documentation, this study explores the potential of AI-generated PVS to serve as a patient-centered communication tool in modern clinical care.

## Methods

### Study Design and Setting

This retrospective study was conducted to assess the readability of PVS generated by an AI-based scribe platform deployed in an orthopedic surgery joint replacement clinic at a regional academic institution. All consecutive patient visits over a 6-month study period from December 2023 to May 2024 were reviewed. A commercial AI-based scribe platform, Abridge Inc, was used to generate all PVS analyzed in this study [19].

### Ethical Considerations

The study protocol was approved by the Medical Center Institutional Review Board prior to initiation (number: 24-05-31-Bada-Read-Vst; approval date: May 31, 2024), and all protocols were conducted in accordance with the Declaration of Helsinki and its amendments [20]. To protect patient privacy, all patient- and provider-identifying information was removed from the dataset before analysis. The AI-generated PVS were anonymized to prevent potential reidentification. The requirement for informed consent was waived by the institutional review board due to the retrospective study design and use of fully deidentified data.

### AI Scribe Patient Visit Process

During each visit, audio recordings of patient-provider conversations were processed using an AI scribe. The system used a large language model–based real-time audio-to-text conversion and AI-driven automatic speech recognition to generate comprehensive visit transcripts. Advanced natural language processing algorithms within the AI scribe software extracted clinically relevant information from transcripts, including patient concerns, symptoms, physical examination findings, diagnoses, treatment plans, and follow-up instructions. The information was then organized into a detailed clinical note with specific sections, such as the history of present illness, physical examination, and assessment and plan.

**Figure 1.**
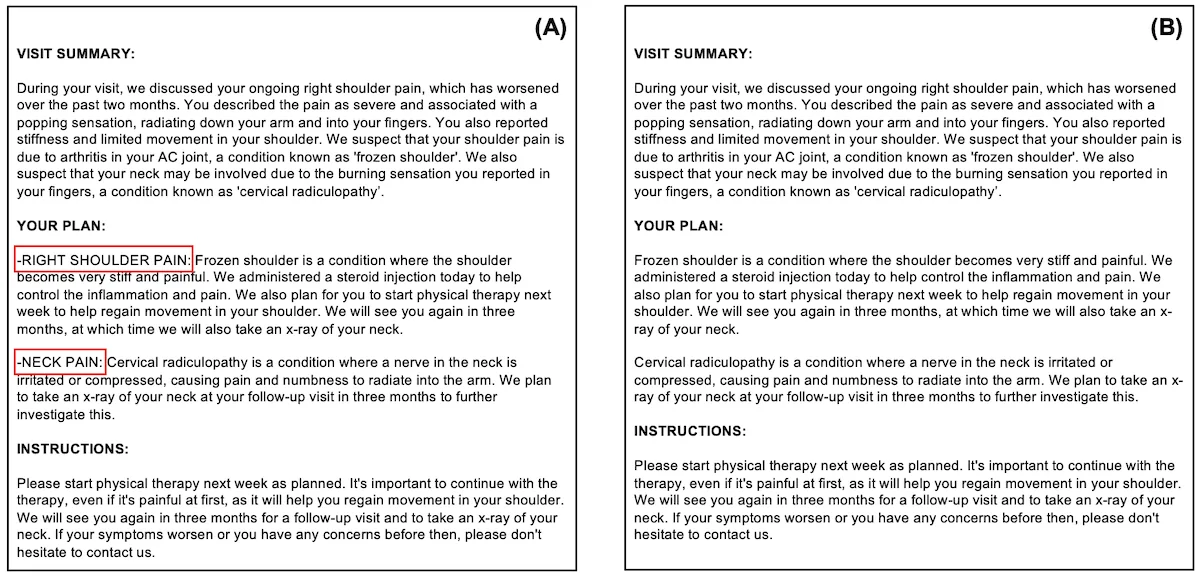
Sample patient visit summary before and after standardized preprocessing. Panel (A) shows the raw AI-generated output, with diagnosis label headers (eg, “-RIGHT SHOULDER PAIN:,” “-NECK PAIN”:) highlighted in red within the “YOUR PLAN” section. Panel (B) shows the same summary following preprocessing, in which diagnosis label headers were removed as they represent structural metadata rather than patient-facing narrative prose. Section headings (“VISIT SUMMARY,” “YOUR PLAN,” and “INSTRUCTIONS”) and all clinical narrative content were preserved without rewriting or simplification. All readability analyses were performed on preprocessed summaries as shown in (B) prior to any provider review or editing.

Using the AI-generated clinical notes, the AI scribe then translated medical jargon into simple language, organized information into clear sections, and integrated the patient’s concerns to generate a personalized, patient-facing PVS for each visit. Providers reviewed all PVS for clinical accuracy prior to release through the patient portal, email, or printout. However, all summaries analyzed in this study reflected the raw, prereview, AI-generated output prior to any provider editing. A sample of an AI-generated PVS is shown in Figure 1.

### Data Collection

A total of 1007 PVS were extracted from the AI scribe platform and converted into plain text for readability analyses. A standardized preprocessing protocol was applied to ensure consistent assessment while preserving patient-facing readability features. The preprocessing workflow included restoration of the original standardized section headings (“VISIT SUMMARY,” “YOUR PLAN,” and “INSTRUCTIONS”) to preserve the intended document organization and formatting generated by the AI scribe. Special characters were removed for software compatibility, including bullet points, degree symbols, and slash characters. Diagnosis label headers (eg, “-RIGHT KNEE PAIN”:) were systematically removed as these labels represented structural metadata rather than narrative patient-facing prose. The details of each diagnosis subheading were preserved as separate paragraph units to maintain the intended structure of the original summaries. Summaries missing one or more major patient-facing sections (“VISIT SUMMARY,” “YOUR PLAN,” and “INSTRUCTIONS”) due to incomplete export or truncation were excluded (25/1007, 2.5%), yielding a final study cohort of 982 summaries.

Following preprocessing, all PVS were uploaded into Readable.com (Readable Ltd), a commercially available readability analysis tool, as plain text and were analyzed using standardized batch text scoring. The original clinical terminology and patient-facing narrative structure were preserved throughout preprocessing to minimize artificial distortion of readability metrics while maintaining the intended patient-facing format [5,6,9,21,22].

### Readability Assessment Methods

The primary readability outcomes were the Flesch-Kincaid Grade Level (FKGL) and the Flesch Reading Ease Score (FRES). FKGL estimates the US grade level required to comprehend a text, with lower scores indicating easier readability. FRES ranges from 0 to 100, with higher scores indicating easier readability and lower scores indicating more difficult readability. FRES categories were interpreted using established classifications: very easy (90-100); easy (80-89); fairly easy (70-79); standard (60-69); fairly difficult (50-59); difficult (30-49); very difficult (0‐29) [21]. Both FKGL and FRES calculate readability based on sentence length (average words per sentence) and word complexity (average syllables per word), with longer sentences and multisyllabic words indicating increased reading difficulty, which is reflected by a higher FKGL and lower FRES.

To compare across multiple metrics, PVS readability was also assessed using the Gunning Fog Index (GFI), Coleman-Liau Index (CLI), and the Simple Measure of Gobbledygook (SMOG) Index [23,24]. The GFI estimates US grade-level readability based on sentence length (average words per sentence) and word complexity (percentage of words with 3 or more syllables), with lower scores indicating better readability. The CLI does not use sentence length but rather calculates readability based on character count per word and word count per sentence, aligning with US grade levels. The SMOG Index, designed for health-related materials, estimates readability by counting polysyllabic words (3 or more syllables) in 30 sentences, with lower values indicating an easier text. Descriptions and formulas of the readability metrics used in this study are summarized in Table 1.

**Table 1.** Commonly used readability scales in health care.

Readability scale	Formula	Range	Interpretation
Flesch-Kincaid Grade Level (FKGL)	Grade level = (0.39 × average # words per sentence) + (11.8 × average # syllables per word) – 15.59	0‐18+	↓ FKGL=easier readability
Flesch Reading Ease Score (FRES)	Reading Ease Score = 206.835 – (1.015 × average # words per sentence) – (84.6 × average # syllables per word)	0‐100	↑ FRES=easier readability
Gunning Fog Index (GFI)	Grade level = 0.4 × [(average # words per sentence) + (# words with >3 syllables) × (100 / # of words)]	0‐20+	↓ GFI=easier readability
Coleman-Liau Index (CLI)	Grade level = (0.0588 × average # of letters per 100 words) – (0.296 × average # of sentences per 100 words) – 15.8	0‐18+	↓ CLI=easier readability
Simple Measure of Gobbledygook (SMOG) Index	Grade level = 1.043 × √ (# of polysyllabic words) × (30 / # of sentences)	0‐18+	↓ SMOG=easier readability

The readability metrics implemented by Readable.com (Readable Ltd), including FKGL, FRES, GFI, CLI, and SMOG, are validated measures commonly used in health communication research [25-28]. Both the AI scribe platform and Readable analysis tool were configured to American English conventions to maintain consistency across all analyzed summaries.

The proportions of PVS with an FKGL at or below the sixth-grade reading level and at or below the eighth-grade reading level were calculated to assess alignment with commonly recommended patient health literacy standards [6] and the average adult reading grade level in the United States [3]. Word count was recorded to evaluate associations between PVS length and readability metrics.

### Data Analysis

The mean, median, SD, range, and 95% CIs were computed for each readability metric to summarize the distribution of readability scores across the analyzed PVS. The Kendall Coefficient of Concordance (*W*) was used to evaluate agreement among the 5 readability metrics in ranking summaries by readability difficulty. Prior to concordance analysis, the FRES was reverse-coded so that higher values consistently represented greater reading difficulty across all readability metrics. Spearman rank correlation (ρ) was used to assess associations between word count and readability metrics due to the nonparametric nature of the correlation analysis. Correlation strength was classified as strong (ρ≥0.7), moderate (0.3≤ρ<0.7), or weak (ρ<0.3), according to established thresholds [29]. All statistical tests were 2-tailed with statistical significance defined as a *P* value of <.05. Statistical analyses were performed using IBM SPSS Statistics (version 31.0; IBM Corp).

## Results

### Study Population

A total of 1007 consecutive AI-generated PVS were identified during the study period, of which 982 met the inclusion criteria for the final analysis.

### Readability of AI-Generated Summaries

**Figure 2.**
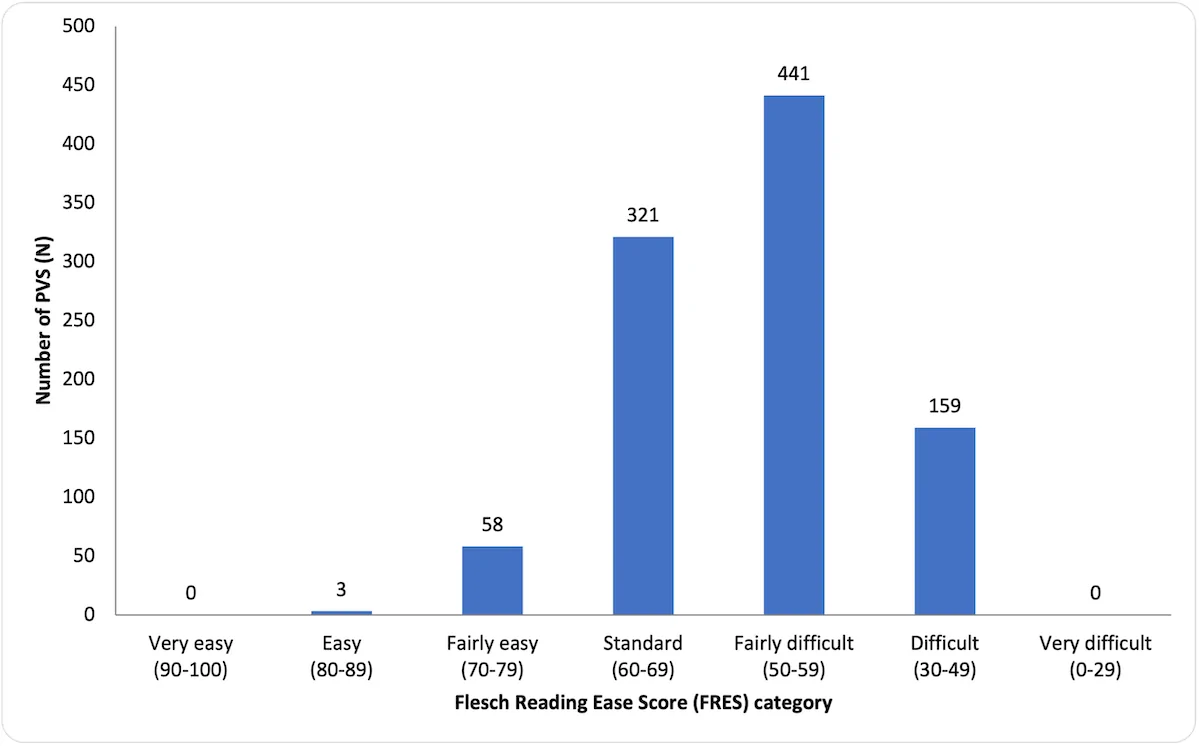
Distribution of patient visit summaries by Flesch Reading Ease Score (FRES) category. This figure displays the distribution of 982 AI-generated patient visit summaries (PVS) across established FRES interpretation categories (range 0-100; higher scores indicate easier readability). Most PVS fell within the “fairly difficult” (441/982, 44.9%) and “standard” (321/982, 32.7%) categories. Smaller proportions were categorized as “difficult” (159/982, 16.2%), “fairly easy” (58/982, 5.9%), and “easy” (3/982, 0.3%). No summaries were classified as “very easy” or “very difficult.”

Across all PVS, the mean FKGL was 9.3 (SD 1.2, 95% CI 9.2‐9.4), with a median of 9.3 and a range from 5.3 to 13.7. The mean FRES was 57.6 (SD 7.9, 95% CI 57.1‐58.1), corresponding to the “fairly difficult” category, with a median of 57.7 and a range of 30.3 to 84.7. The distribution of PVS across FRES categories is shown in Figure 2.

The GFI demonstrated a mean score of 12.4 (SD 1.6, 95% CI 12.3‐12.5), ranging from 6.8 to 18.8. The CLI demonstrated a mean grade level of 11.0 (SD 1.5, 95% CI 11.0‐11.1), with a range of 6.1 to 16.0. The SMOG Index demonstrated a mean grade level of 12.4 (SD 1.2, 95% CI 12.3‐12.4), with scores ranging from 7.7 to 16.5. Comprehensive descriptive statistics for all readability indices are presented in Table 2.

**Table 2.** Readability summary statistics for AI-generated patient visit summaries.

Readability index	Mean (SD)	Median	Range	95% CI
Flesch-Kincaid Grade Level (FKGL); range: 0‐18+ (↓=better)	9.3 (1.2)	9.3	5.3‐13.7	9.2‐9.4
Flesch Reading Ease Score (FRES); range: 0‐100 (↑=better)	57.6 (7.9)	57.7	30.3‐84.7	57.1‐58.1
Gunning Fog Index (GFI); range: 0‐20+ (↓=better)	12.4 (1.6)	12.3	6.8‐18.8	12.3‐12.5
Coleman-Liau Index (CLI); range: 0‐18+ (↓=better)	11.0 (1.5)	11.1	6.1‐16.0	11.0‐11.1
SMOG Index; range: 0‐18+ (↓=better)	12.4 (1.2)	12.4	7.7‐16.5	12.3‐12.4

**Figure 3.**
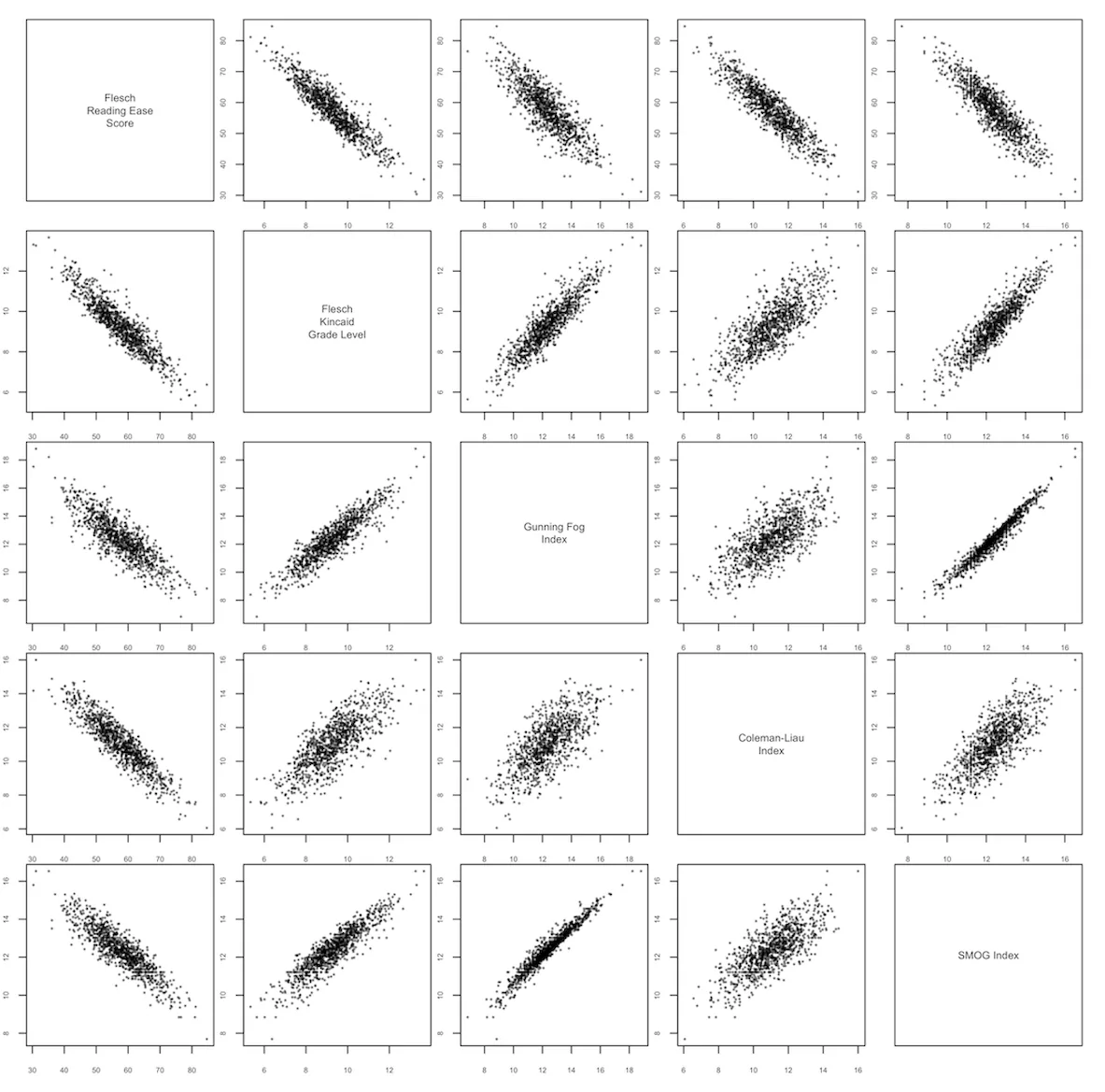
Scatter plot matrix illustrating pairwise relationships among 5 readability metrics for 982 AI-generated patient visit summaries. Strong linear relationships were observed across all metric pairs, consistent with high intermetric concordance.

Kendall Coefficient of Concordance (*W*) analysis demonstrated strong agreement in readability rankings across the 5 metrics (*W*=0.882, *P*<.001). The pairwise associations shown in Figure 3 were consistent with this high degree of intermetric concordance.

### Alignment With National Literacy Benchmarks

**Figure 4.**
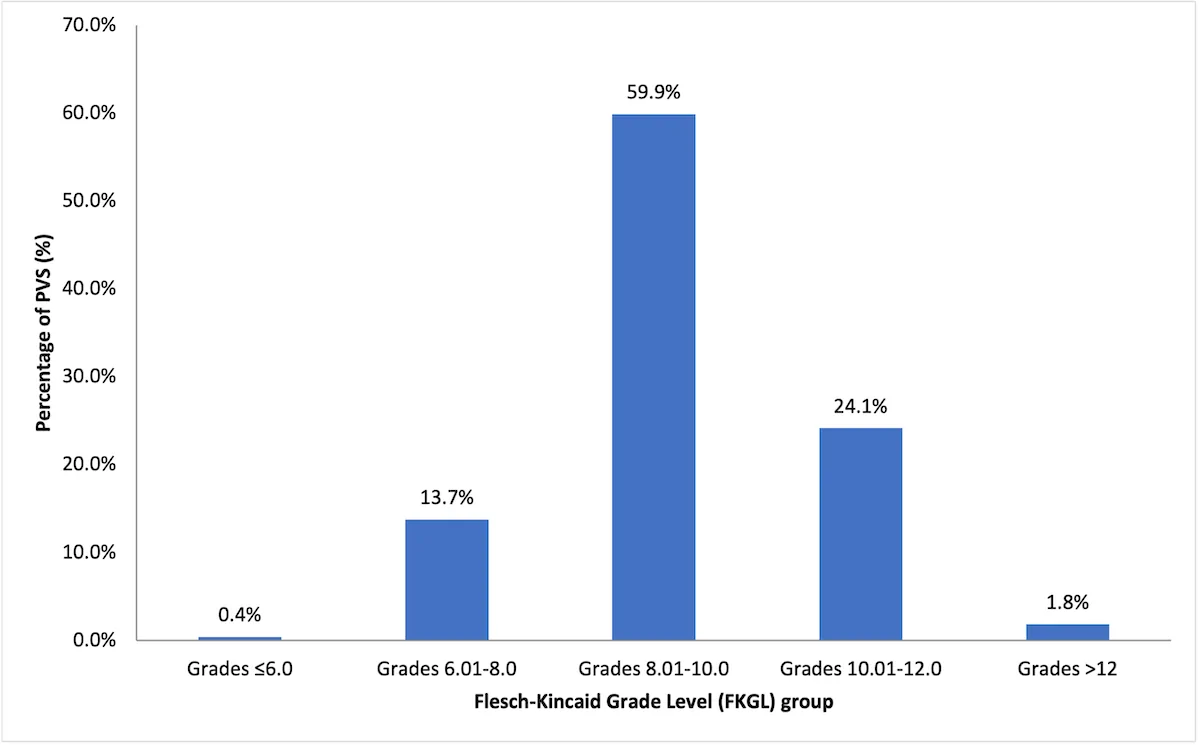
Distribution of patient visit summary readability by Flesch-Kincaid Grade Level (FKGL). This figure displays the percentage distribution of AI-generated patient visit summaries (PVS) across FKGL categories. Most summaries (588/982, 59.9%) fell within the grade 8.01‐10.0 range, whereas 237 (24.1%) were within grades 10.01‐12.0, and 135 (13.7%) were within grades 6.01‐8.0. Only 4 summaries (0.4%) met the sixth-grade benchmark, whereas 18 (1.8%) exceeded grade 12.

Only 0.4% (4/982) of PVS met the recommended sixth-grade reading level threshold for health communication materials. A higher proportion of 14.2% (n=139) of PVS met the eighth-grade benchmark. The remaining summaries were distributed across higher FKGL ranges: 588 (59.9%) between grades 8.01‐10.0, 237 (24.1%) between grades 10.01‐12.0, and 18 (1.8%) greater than grade 12. Figure 4 illustrates the cumulative percentage of summaries that met the various grade-level thresholds.

### Relationship Between Word Count and Readability

The mean word count per PVS was 202.0 (SD 47.0, median 200.0, range 84.0‐389.0, 95% CI 199.1‐205.0). Spearman rank correlations between word count and readability metrics were uniformly weak (|ρ|<0.15). Statistically significant but negligible correlations were identified for GFI (ρ=0.082, *P*=.01) and CLI (ρ=−0.111, *P*<.001). Word count was not significantly correlated with FKGL (ρ=0.012, *P*=.70), FRES (ρ=0.032, *P*=.320), or the SMOG Index (ρ=0.027, *P*=.40). These findings suggest that summary length alone did not meaningfully determine readability level within this cohort. The complete correlation results are summarized in Table 3.

**Table 3.** Spearman rho correlation of word count with readability scores.

Readability scale	Spearman rho coefficient (ρ)	*P* value	95% CI
Flesch-Kincaid Grade Level	0.012	.70	−0.054 to 0.071
Flesch Reading Ease Score	0.032	.32	−0.037 to 0.108
Gunning Fog Index	0.082	.01	0.020 to 0.137
Coleman Liau Index	−0.111	<.001	−0.174 to −0.049
SMOG Index	0.027	.40	−0.037 to 0.085

## Discussion

### Principal Findings

Effective patient-provider communication is a cornerstone of quality health care, particularly in specialized fields such as orthopedic surgery, where the complexity of information can often be overwhelming. This study evaluated 982 AI-generated PVS and found an average readability corresponding to a ninth-grade reading level. Only 0.4% (4/982) of summaries met the sixth-grade threshold and 14.2% (139/982) met the eighth-grade benchmark. In the absence of a comparison group, conclusions regarding relative improvement over traditional methods could not be established. These findings establish a quantitative readability baseline for AI scribe–generated PVS in an orthopedic setting and highlight the need for algorithmic refinement to better align patient-facing outputs with recommended health literacy standards.

The mean FKGL of 9.3 aligns with prior studies, showing that most orthopedic educational materials exceed national literacy recommendations. For example, Karimi et al [8] reported FKGL scores ranging from 10 to 13 in total joint arthroplasty education materials, while Sahhar et al [3] found that 100% and 76.3% of preoperative orthopedic education articles exceeded sixth- and eighth-grade reading levels, respectively. Similarly, Gerhold et al [30] reported that online orthopedic trauma materials had an average FKGL of 8.7, with only 1.25% at or below a sixth-grade level. These findings collectively illustrate that even modest deviations from national readability standards can limit comprehension, satisfaction, and engagement [3,5].

Beyond orthopedics, previous analyses of electronic health record-generated or online patient education materials across medical specialties (eg, internal medicine, cardiology, and radiology) have identified similar mismatches between readability and average US literacy levels, suggesting that this is a system-wide issue rather than specialty-specific [31-33]. By contextualizing AI-generated PVS within this broader landscape, this study contributes baseline data on the readability performance of AI-generated documentation relative to existing patient-facing materials.

Spearman correlations between word count and readability metrics were uniformly weak (|ρ|<0.15) across all 5 indices. Although GFI (ρ=0.082, *P*=.01) and CLI (ρ=−0.111, *P*<.001) reached statistical significance, FKGL, FRES, and SMOG did not. The small effect sizes observed across all indices suggest that statistical significance in GFI and CLI likely reflected the large sample size rather than a clinically meaningful relationship. These findings suggest that sentence structure and vocabulary complexity, rather than overall summary length, were the primary drivers of readability in AI-generated PVS.

Beyond readability scores, limited health literacy has been consistently linked to worse surgical outcomes, higher health care utilization, and reduced adherence [3,5]. Inadequately readable summaries risk compounding these issues, as comprehension is essential for informed consent, medication adherence, and effective postoperative care.

AI scribe platforms represent a promising digital health intervention that could bridge these gaps. Unlike static templates, generative AI can be tailored to match a patient’s literacy level, potentially generating multiple versions of a summary based on individual needs. This adaptability addresses the long-standing limitations of traditional visit summaries and autogenerated materials [17]. Emerging AI platforms, including the one evaluated in this study, continue to evolve and have the potential to incorporate real-time feedback loops that dynamically adapt readability. Optimizing these systems with plain language frameworks, algorithmic adjustments, and targeted prompting strategies may enhance comprehension without sacrificing accuracy. The finding that 14.2% of summaries already met the eighth-grade threshold compared with only 0.4% meeting the sixth-grade standard suggests that a tiered optimization approach targeting the eighth-grade benchmark first may represent a more achievable near-term goal. This is particularly relevant given that specialty-specific medical terminology may inherently constrain readability. However, any efforts to improve readability must not compromise clinical accuracy. AI-generated content carries an inherent risk of factual errors and hallucinations, and readability optimization strategies must be validated to ensure language simplification does not introduce clinically unsafe inaccuracies. The balance between language simplification and preservation of medical precision remains a critical design challenge that must be validated through further patient-centered testing in the future.

### Strengths and Limitations

This study had several strengths, including its large sample size, focus on a specific clinical context, and the use of multiple established readability formulas to benchmark AI-generated PVS. However, this study has several limitations that should be considered.

First, traditional readability formulas are deterministic, surface-level metrics designed for static human-authored text. AI-generated content carries additional interpretive limitations. A summary may achieve a favorable FKGL through short sentences while remaining clinically incoherent, and a high grade level does not in itself indicate an inaccurate summary. The strong concordance across 5 indices (*W*=0.882, *P*<.001) confirms consistent rank ordering of surface linguistic complexity rather than semantic coherence, clinical fidelity, or actual patient comprehension. Future research should incorporate validated qualitative instruments, such as the Patient Education Materials Assessment Tool and DISCERN tool, to evaluate comprehensibility and accuracy beyond surface readability metrics.

Second, the absence of real patients in this study limited the ability to draw conclusions about the practical impact of AI-generated summaries on patient understanding. The retrospective design precluded the collection of comprehension data or usability feedback, and the findings represent a single point in time that may evolve as AI scribe technology improves.

As all analyzed summaries reflected the raw, prereview AI output, the cognitive burden of simplifying a mean FKGL of 9.3 to the recommended sixth-grade level standard falls on the reviewing provider, a task that may partially offset the time-saving premise of AI scribes.

Third, the absence of demographic data such as education level, language proficiency, and health literacy hindered the evaluation of how patient characteristics affect summary complexity. This analysis was conducted in an academic joint replacement clinic, potentially introducing a selection bias. In this context, patients may possess higher health literacy or educational levels compared to the general population, which may limit the generalizability of our findings to other populations. However, prior studies have reported that orthopedic patients generally demonstrate lower health literacy than broader medical populations, suggesting that readability barriers may persist despite the academic setting [8]. Additionally, academic centers typically manage higher complexity cases compared to community practices, potentially introducing more specialized terminology and inflation of measured readability relative to a community orthopedic setting. Fourth, the evaluation of a single commercial AI scribe system without readability-optimized prompting limits generalizability to other models or institutions [9]. Although this allowed for a real-world evaluation of the platform’s baseline output, we recognize that AI systems can be tailored through prompting or algorithmic adjustments to produce content that better aligns with national readability standards, as demonstrated in previous studies [9]. Finally, the lack of a preimplementation baseline using physician-generated visit summaries restricted direct comparisons. There are no previous studies evaluating the readability of visit summaries in orthopedic surgery, as the only published benchmark for physician-generated visit summaries was conducted in the fields of internal medicine and family medicine [31], which are not comparable to orthopedic surgery settings.

Future research should incorporate patient-reported outcomes and comprehension testing to assess whether enhanced readability leads to tangible improvements in understanding and adherence. Comparative evaluations across multiple generative AI systems, with and without readability optimization, are required to assess platform-level variability. Future investigations should focus on developing advanced readability scales that integrate semantic and contextual understanding, rather than depending solely on sentence structure. Future trials should examine how AI-generated summaries affect patient engagement, provider workload, and the quality of documentation. Finally, ongoing reevaluation of the sixth- and eighth-grade benchmarks is necessary to determine whether these standards remain appropriate for contemporary digital health communication.

### Conclusions

In this orthopedic outpatient setting, AI-generated PVS consistently exceeded recommended patient literacy standards, with a mean FKGL of 9.3 and only 14.2% of summaries meeting the eighth-grade reading standard. In the absence of a contemporaneous comparison group, conclusions regarding relative improvement over traditional methods could not be established. Future studies should evaluate whether tailored strategies, patient comprehension testing, and multiplatform comparisons can further enhance readability and improve health communication.

## Abbreviations

CLI: Coleman-Liau Index
FKGL: Flesch-Kincaid Grade Level
FRES: Flesch Reading Ease Score
GFI: Gunning Fog Index
PVS: patient visit summary
SMOG: Simple Measure of Gobbledygook
